# The Effect of Annealing Treatment and Atom Layer Deposition to Au/Pt Nanoparticles-Decorated TiO_2_ Nanorods as Photocatalysts

**DOI:** 10.3390/molecules23030525

**Published:** 2018-02-09

**Authors:** Shuang Shuang, Zhengjun Zhang

**Affiliations:** 1 State Key Laboratory of New Ceramics and Fine Processing, School of Materials Science and Engineering, Tsinghua University, Beijing 100084, China; shuangshuang_buct@163.com; 2 Key Laboratory of Advanced Materials (MOE), School of Materials Science and Engineering, Tsinghua University, Beijing 100084, China

**Keywords:** TiO_2_ NPAs, atomic layer deposition, Au/Pt NPs, photoelectrochemistry, visible light

## Abstract

The wide band gap of TiO_2_ hinders the utilization of visible light in high-performance photocatalysis. Herein, vertically aligned Ti nanopillar arrays (NPAs) were grown by the glancing angle deposition method (GLAD) and then thermally oxidized into TiO_2_ NPAs. The metallic nanoparticles (NPs) were fabricated by successive ion layer adsorption and reaction (SILAR) method. And we covered ultrathin TiO_2_ layer on Au/Pt NPs decorated NPA using atomic layer deposition (ALD) method and did annealing process in the end. The photoelectrochemical (PEC) performance and dye degradation have been studied. We find the dye degradation efficiency of best combination reaches up to 1.5 times higher than that of original Au/Pt-TiO_2_ sample under visible light irradiation. The TiO_2_ ALD layer effectively protects the nanostructure from corrosion and helps the transmission of electrons to the electrolyte. By controlling the annealing temperature we could achieve a matched band gap due to change in noble metal particle size. Our work demonstrates that rational design of composite nanostructures enhances the usage of broader wavelength range light and optimizes photocatalytic degradation of organic pollutants in practical applications.

## 1. Introduction

Composite nanomaterials have attracted researchers’ attention due to their multifunctional applications in various fields, such as semiconductor photocatalysts for pollutants degradation. TiO_2_, due to its nontoxic nature, long-term photostability, low cost, and relatively efficient photocatalytic activity, has been investigated in this context for many years [1]. However, it is just effective and operative in the UV region because of its wide band gap (~3.0 eV) [2] and the UV region only accounts for about ~4% in the solar radiation [3]. Moreover, the photogenerated electron-hole pairs have a quite short recombination time that also hinders further utilization of TiO_2_. Previously, a lot of work has been done to address these issues with apporaches such as noble-metal decoration [4], ion doping [5,6,7] or alteration of the unit morphology [8]. Nowadays the quantum size effect has been focused on as another hot topic [9,10]. TiO_2_ nanostructures tend to aggregate easily, which effects the efficiency in practical experiments. Here we use the glancing angle deposition (GLAD) technique to fabricate TiO_2_ nanopillar arrays (NPAs) vertically on substrates which are conveniently recycled and guarantee enough reaction surface area during the whole process.

Nanostructured noble metals could have a collective coherent oscillation of surface electrons which enables noble metals nanoparticles (NPs) to present surface plasmon resonance (SPR) effects [11,12]. These kinds of nanostructures show enhanced performance in a broad light range including the visible as well as the UV region [13,14,15], so TiO_2_ is usually combined with other semiconductors to form composites which possess certain functions such as photocatalytic properties. Li et al. [16] have designed a CdS-Au-TiO_2_ sandwich nanorod array capable of producing a photocurrent of 4.07 mA·cm^−2^ at 0 V (vs. Ag|AgCl) under full solar spectrum irradiation. It also showed a maximum solar-to-chemical energy conversion efficiency of 2.8%, which is much higher than that of pure TiO_2_. Herein Au NPs serve as plasmonic photosensitizers and form a Schottky barrier with TiO_2_. CdS and TiO_2_ are band gap matched, which enhances the excited separation of electrons and holes. As for the synthesis of noble metal particles on nanostructures, there are many ways to achieve this such as chemical vapour deposition [17], sol-gel synthesis [18], hydrothermal methods [19] and successive ion layer adsorption and reaction (SILAR) [20]. Here we have co-decorated both Au and Pt NPs on the surface of nanorods through the SILAR method because of the easy preparation in room temperature.

Corrosion always happens on nanostructures during the degradation process [21]. In order to protect nanostructures and suppress the recombination, a metal oxide thin layer such as Al_2_O_3_ [22], SnO_2_ [23] and ZrO_2_ [24] is always applied through atomic layer deposition (ALD) or other methods [25,26]. George et al. have employed Al_2_O_3_ and TiO_2_ ALD when fabricating an ultrathin barrier film on a Cu substrate to prevent water corrosion [27]. As for catalysis, Lu et al. [28] have applied a TiO_2_ overcoat on Au catalysts by ALD. They found higher activity in CO oxidation and investigated the reaction mechanism. This technique could control the thickness of layers at the nanoscale by setting the number of deposition cycles and self-limiting the reaction of dosed precursor gases.

**Figure 1:**
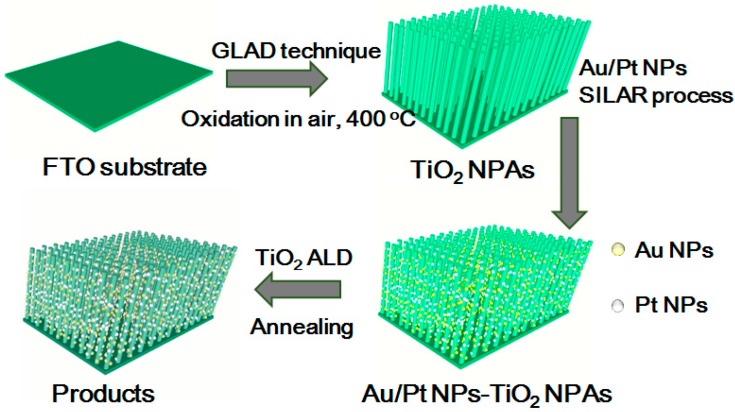
Schematic illustration of the preparation of Au/Pt-TiO_2_ nanorod arrays (NPAs) with ALD and the annealing treatment.

Herein, we report co-decorated Au/Pt NPs on TiO_2_ NPAs with a wrapped up TiO_2_ ALD layer. TiO_2_ nanopillar arrays (NPAs) were first deposited on SiO_2_, F-doped SnO_2_ (FTO) and Si substrates through the GLAD method. Later metallic NPs (Au and Pt) were deposited on the NPAs using the SILAR method [29]. Then, we coated a TiO_2_ ALD layer on the nanostructured material. In order to improve the stability of the photocatalyst, we annealed the sample at different temperatures. The whole process is shown in Figure 1. We have also studied the effect of annealing on the photocatalytic performance of our fabricated nanostructures.

## 2. Results and Discussion

### 2.1. Characterization of Materials

**Figure 2:**
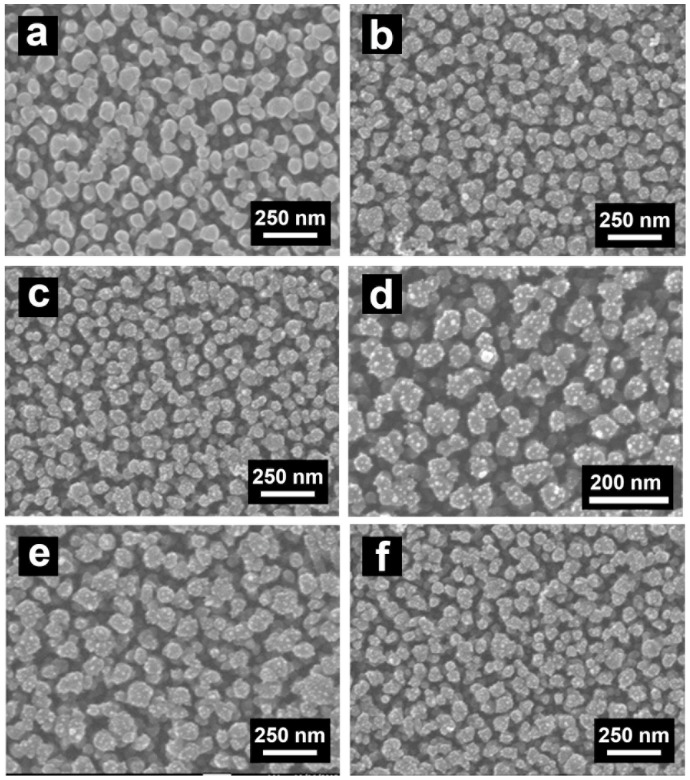
SEM images of: (**a**) TiO_2_ NPAs; (**b**) Au/Pt-TiO_2_ NPAs; (**c**) Au/Pt-TiO_2_ NPAs coated with one ALD layer; (**d**) magnified image of Au/Pt-TiO_2_ NPAs coated with one ALD layer, Au/Pt-TiO_2_ NPAs coated with three (**e**); or four (**f**) ALD layers.

Figure 2 presents the SEM images of pure TiO_2_ NPAs (Figure 2a), coated only with noble metal NPs (Figure 2b), Au/Pt-TiO_2_ NPAs coated with one, three, and four cycles of TiO_2_ ALD (Figure 2c,e,f), and a magnified image of Au/Pt-TiO_2_ NPAs coated with one cycle of TiO_2_ ALD (Figure 2d), respectively. From the top view we can see that the Au and Pt nanoparticles are distributed on the TiO_2_ NPAs’ surface (Figure 2b), where they appear as little white dots randomly spread on the TiO_2_ NPA surface. It is difficult to distinguish the modifications in the nanostructures only from the morphology, because ALD application and the annealing treatment do not change them remarkably.

**Figure 3:**
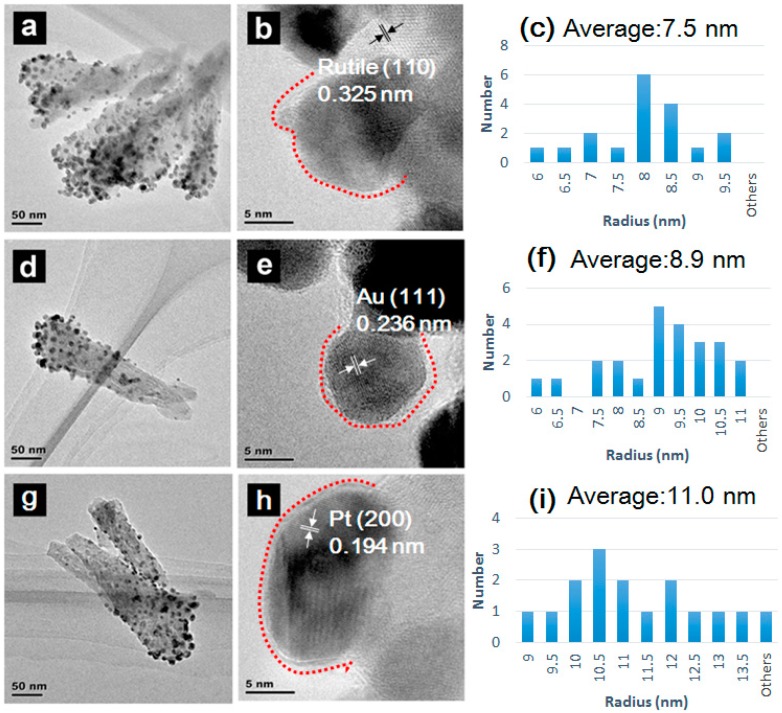
TEM images, HRTEM images and particles size statistics of (**a**–**c**) 2ALD/Au/Pt-TiO_2_ NPAs; (**d**–**f**) 2ALD/Au/Pt-TiO_2_ NPAs annealing at 200 °C; (**g**–**i**) 2ALD/Au/Pt-TiO_2_ NPAs annealing at 300 °C.

In order to improve the degree of crystallinity, we tried anneal the samples after ALD coating. Here we choose three different temperature (200 °C, 300 °C and 400 °C, respectively). The TEM images in Figure 3 show TiO_2_ without annealing (Figure 3a,b), 300 °C (Figure 3d,e) and 400 °C (Figure 3g,h) annealing based on two cycles of ALD TiO_2_. The TiO_2_ NPAs have a size of approximately 200 nm in length and a diameter of about 50 nm (Figure 3a). The calculation and test of lattice fringes, (d = 0.325 nm, 0.236 nm and 0.194 nm) matches with the lattice plane of rutile (110), Au (111) and Pt (111), respectively [30]. The generation of metal-semiconductor nanojunctions can result in the formation of a Schottky junction, which can dramatically affect the optoelectronic properties of the material. When Au and Pt contact to TiO_2_, the charge carriers would redistribute. Electrons move from the n-type semiconductor (higher Fermi level) to the metal (lower Fermi level) until reaching equilibrium. The Schottky barrier which could efficiently capture excited electrons. These facts would all be favourable to interfacial charge transfer between each component and improve the photocatalytic performance of the whole system eventually [31]. Our experimental results show that the interface of ALD thin film becomes clearer with higher crystallinity. This phenomenon indicates that the degree of crystallinity of our nanostructures improves with annealing treatment [32]. Here the number of ALD cycles is indicated before the term “ALD” in the sample name.

**Figure 4:**
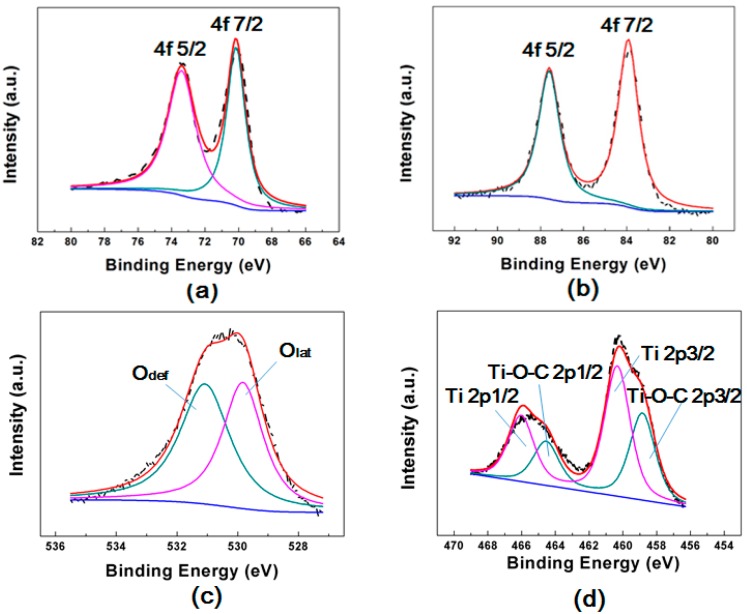
XPS spectrum of 2ALD/Au/Pt-TiO_2_ NPAs: (**a**) Pt 4f; (**b**) Au 4f; (**c**) O 1s; (**d**) Ti 2p.

To confirm the valence of metallic NPs, the 2ALD/Au/Pt-TiO_2_ NPAs without annealing sample was investigated by the XPS technique (Figure 4). The intense doublet of Pt (70.2 and 73.6 eV) and Au (83.7 and 87.4 eV) correspond to metallic Pt^0^, Au^0^ [33]. These results show that the Au and Pt NPs were both successfully fabricated on TiO_2_ NPAs. Furthermore the outer layer is also TiO_2_. The O 1s XPS spectrum (Figure 4c) can be fitted by two peaks: a main peak showing at a binding energy of 529.8 eV which is attributed to lattice ‘O’ and another one located at 531.4 eV that refers to the hydroxyls or water adsorbed on the surface of the nanostructure. The Ti 2p spectrum includes doublets of Ti 2p_1/2_ and Ti 2p_3/2_ at binding energies of 466.1 and 460.3 eV which confirm the existence of Ti^4+^ cations in TiO_2_ [34]. There are two peak located around 464.6 and 458.8 eV indicating Ti-O-C 2p_1/2_ and Ti-O-C 2p_3/2_ bonds which are due to the ALD reaction process which indicates intermediate products formed after the reaction between tetrakis(dimethylamido)titanium(IV) and H_2_O [35].

**Figure 5:**
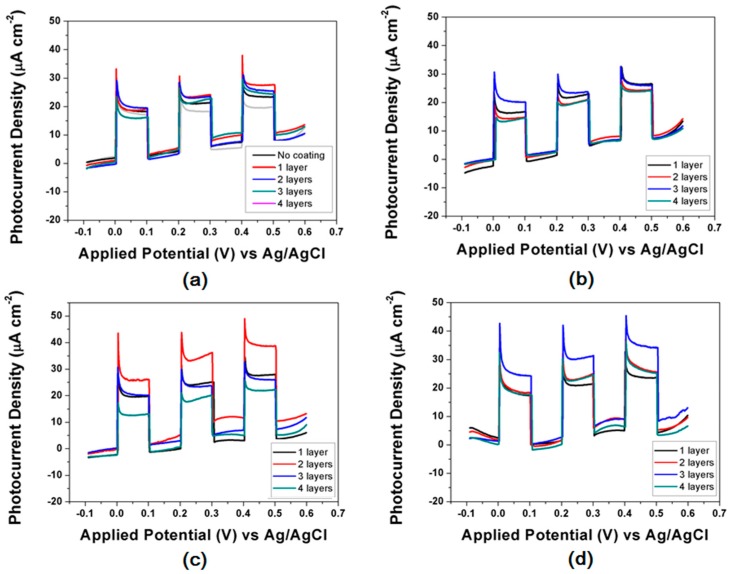
*j-V* characteristics (with light on/off) of (**a**) Au/Pt-TiO_2_ NPAs and coated with 1~4 ALD layers; Au/Pt-TiO_2_ NPAs coated with 1~4 ALD layers under 200 °C (**b**); 300 °C (**c**); 400 °C (**d**) annealing treatment.

Figure 5 show on-off PEC plots for Au/Pt-TiO_2_ NPAs with ALD coatings (1, 2, 3, 4 layers) and the corresponding annealed samples’ test result. The figures compare the photocurrent density of the series of samples with increased potential from −0.1 to +0.6 V. According to Figure 5a,b, we can see that the currents between light on/off are all about 15 μA cm^−2^, which doesn’t change much even under 200 °C heat treatment. However, when annealed at 300 °C and 400 °C for 1 h, the photocurrent generally increased. Especially, 300 °C annealing with two ALD layer cycles and 400 °C with three layers reach to 40 and 35 μA cm^−2^ (Figure 5c,d). They are enlarged about 1.5~2 times compared to the previous one without annealing treatment.

We also investigated the photocatalytic performance and degradation of Methylene Blue (MB) dye under visible light for 1 h. The Au/Pt-TiO_2_ NPs sample shows a degradation efficiency of 49.3%. Furthermore we analyzed the degradation efficiency of our composite nanostructures with different ALD cycle numbers and annealing temperature, as mentioned in Table 1. Our experiments show that after ALD coating and heat treatment most samples showed an improvement in the photocatalytic degradation. The sample without ALD shows lower efficiency because the metal NPs are easily photocorroded without the protection of the outer coating layer [36]. Compared with the ALD-coated samples, the excited electrons could not only transfer through TiO_2_ NPAs, but also ALD TiO_2_ which surrounds the metal NPs. Specifically the 2ALD/Au/Pt-TiO_2_ NPAs sample (with annealing at 300 °C) shows the highest degradation efficiency which is enhanced by about 150% compared with the blank sample. However, on the contrary the efficiency of composite nanostructures annealed at 400 °C goes down. For samples with the same annealing temperature, two cycles and three cycles usually show better performance. Through our experiemntal data calculations, annealing mainly influences and enhances the degradation efficiency of the MB pollutant. Usually demethylation would happen on MB when degraded by TiO_2_, thus the absorption peak of the dye solution will cause a blue shift [37].

In order to study the effect of annealing treatment in depth, diffuse reflectance UV-vis spectra of five different products (TiO_2 _NPAs, Au/Pt-TiO_2_ NPAs and Au/Pt-TiO_2_ NPAs with annealing for 200 °C, 300 °C, 400 °C) are presented in Figure S1, which are also calculated with the Kubelka−Munk function. The equation of the band gap energy (E_g_) is ${\alpha E}_{\mathrm{photon}}=K{\left(E_{\mathrm{photon}}-E_{g}\right)}^{1/2}$, where α, K, E_g_ and E_photon_ are respectively the absorption coefficient, a constant, the band gap energy and the discrete photon energy. Here we plot and calculate the intercept of an extrapolated linear fit [20].

TiO_2_ NPAs exhibit a UV absorption peak at 300~400 nm. The metal decoration of TiO_2_ NPAs indicates a remarkable improvement in the light absorption at about 480 nm due to the surface plasmon absorption of the decorated Au NPs [38], but we didn’t find any obvious difference in the UV adsorption after the annealing treatments, thus we turned our attention to the size of the metal NPs. Here we compare the statistical NPs radius according to the TEM images. The histograms are plotted in Figure 3c,f,i. From the average diameter statistics, the size of 2ALD/Au/Pt-TiO_2_ NPAs without annealing and annealing under 300 °C and 400 °C increases with higher heat treatment from 7.5 nm to 11.0 nm, but we could find that there is an extremum in the photoccatalytic properties, which means there probably exists an optimal NP diameter size.

### 2.2. Photocatalytic Mechanism

After visible light irradiation, strong SPR is excited by Au NPs [39]. During its dephasing, hot electrons are injected via Schottky junction into the conduction band (CB) for reduction reactions, leaving holes in the noble metal nanoparticles where oxidation reactions occur [40,41,42]. So in this system hot electrons shift from the surface of the Au NPs to the TiO_2_ conduction band [43]. The work function of Pt (~5.40 eV in vacuum [44]) is higher than that of Au (~4.78 eV in vacuum) which leads to the Fermi level of Pt being lower than that of Au. Thus electrons would move from Au to Pt via TiO_2_. Here Pt NPs play a role as cocatalyst. 

**Figure 6:**
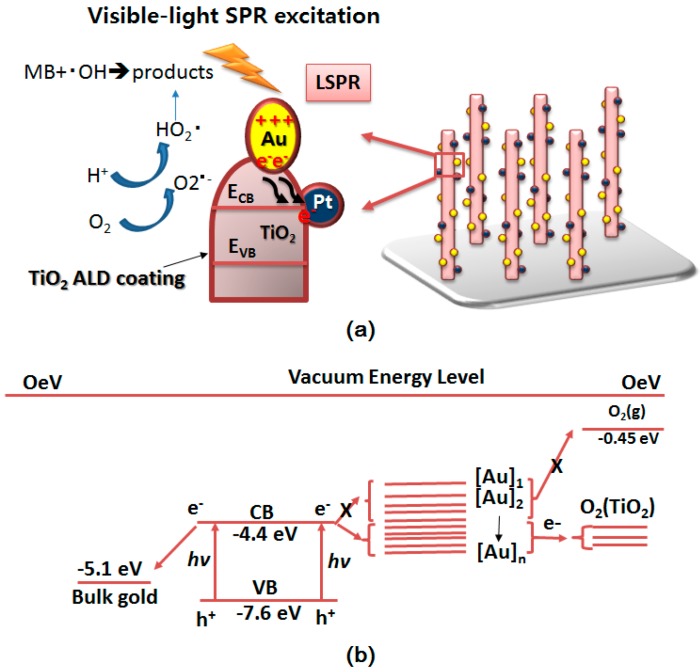
(**a**) The charge transfer process of TiO_2_ ALD/Au/Pt/TiO_2_ NPAs under UV-vis lights; (**b**) Fermi level of nanoscale gold particles.

Here electrons can both decompose the dye and interact with electron acceptors (O_2_) to create superoxide radicals (O_2_^•−^). At same time, the positively charged Au NPs would combine with OH^−^ into highly oxidizing species such as hydroxyl radicals, OH• or oxidize the MB molecule directly. The whole reaction is presented in Figure 6a. It is necessary to be aware that a close Au/TiO_2_ Schottky contact is due to the last-step annealing process. The treatment is helpful to produce an efficient visible-light photocatalyst [45]. Furthermore heterojunction arrays, which is the light trapping caused from the one-dimensional array nanostructure with matched bandgap, also improve the light absorption [46].

When the size reaches the nanoscale, the Fermi level of noble metal NPs usually improves with decreased size because of the quantum size effect. The mechanism is shown in Figure 6b. A suitable diameter could be achieved to match the band gap. Only this could convert electrons efficiently and suppress the combination of electrons and holes [47,48]. The energy level of Au NPs must stay higher than that of oxygen gas and lower than the CB of TiO_2_. When hot electrons are excited by visible light from Au NPs, just the rational energy level could let electrons move into the CB of TiO_2_ and be able to react with O_2_ to form O_2_^•−^. Otherwise, if the energy of the Au NPs becomes too high, it won’t transfer to TiO_2_; or if the energy becomes too low, it won’t have enough energy to react with O_2_. What’s more, if the particles become too big, they will also cover too much of the TiO_2_’s reaction surface which in turn hinders the photocatalytic properties.

## 3. Materials and Methods

### 3.1. Synthesis of TiO_2_ NPAs

Firstly, Ti NPAs were fabricated via e-beam using the GLAD technique on several substrates (i.e., quartz, silicon and FTO). The chamber need to be evacuated to 1 × 10^−8^ Torr before deposition. All substrates were cleaned using ultrasonication in acetone, ethanol and deionized (DI) water, respectively. The data of deposition was similar to that used in previous work [49]. After deposition, the thin films were oxidized in a tube furnace at 400 °C for 2 h.

### 3.2. Metallic Nanoparticle Decoration on TiO_2_ NPAs

Au and Pt NPs were reacted via the SILAR method with some modifications as previously published [49]. We dipped TiO_2_ successively into HAuCl_4_ (or HPt_2_Cl_6_) and reductant (NaBH_4_) solutions in order to form NPs. The substrates were immersed into a certain concentration of HAuCl_4_ (or HPt_2_Cl_6_) solution, DI water, 1 mg/mL NaBH_4_ solution, DI water respectively for 60 s. This complete procedure is regarded as one cycle. The whole process was repeated until the desired cycle number was fabricated. Au/Pt-TiO_2_ NPAs samples were consecutively deposited by Au and Pt NPs individually five times.

### 3.3. ALD Deposition

TiO_2_ NPAs were placed in a custom-built reactor, and the substrate temperature was kept at 150 °C during the ALD process [50]. When pumped into the chamber, the precursor, tetrakis(dimethylamido)titanium(IV), was controlled at 110 °C and water was at 40 °C. A soaking step was inserted in order to confirm the sufficient diffusion of the reacting molecules on the surface of the nanostructures [51]. The precursor was first pumped using 200 ms and soaked for 5 s, after which the chamber was evacuated for 20 s. Later, H_2_O (gas) was pulsed for 5 ms and kept for 3 s to let them react, with a 20 s purge step at the end. This whole procedure can be regarded as one ALD cycle. The process is repeated until the desired thickness of TiO_2_ is achieved.

### 3.4. Characterization

The chemical nature, morphology and nanostructure of all samples were characterized by field-emission scanning electron microscopy (SEM, FE-SEM, JEOL-7001F, JEOL, Tokyo, Japan) and high-resolution transmission electron microscope (HRTEM, JEOL-2011, JEOL). The surface composition was determined by X-ray photoemission spectroscopy (XPS) measurements performed on a PHI 5300 instrument (Perkin Elmer, Waltham, MA, USA). Here the binding energy was calibrated with the C1s peak (284.6 eV). The light absorption of materials were tested via a Perkin Elmer Lambda 35 UV-Vis spectrometer (Perkin Elmer) in the wavelength range between 375 nm and 900 nm.

### 3.5. Property Measurements

The photocurrent density was measured by an electrochemistry workstation (CHI 660D, Chenhua Instrument, Shanghai, China). Here the film and substrates were regarded as the working electrode, while a Ag/AgCl electrode (saturated KCl) is used as a reference. Pt slices with specific shapes were applied as counter electrodes. A Xe lamp (300 W) was used as a light source with ultraviolet filter to intercept the UV light (wavelength shorter than 420 nm). Photocurrent densities with increased potential from −0.1~+0.6 V bias vs Ag/AgCl electrode were tested via a light on-off process. Here we selected intervals of 5 s for the switching time. The photocatalytic degradation performance was examined by using Methylene Blue (MB) under visible light. The samples on a quartz substrate (15 mm × 15 mm) were immersed in a 25 mL beaker containing 5 mL of MB (5 μM) solution. The samples remained in solution for half an hour before degradation to achieve an adsorption/desorption equilibrium. At the end of 1 h irradiation, we tested the concentration of MB by UV-Vis spectroscopy at its characteristic wavelength of 502 nm. Both remaining percentage (C/C_0_) and degradation efficiency (1 − C/C_0_) × 100% were recorded. PEC experiments with light on-off at specific voltages were also performed to assess the photoelectrochemical properties. 0.1 M Na_2_SO_4_ aqueous solution was used as the electrolyte.

## 4. Conclusions

In conclusion, we have successfully prepared TiO_2_ ALD/Au/Pt NPs-decorated TiO_2_ nanostructures with an annealing treatment. When compared with nanostructures without ALD and annealing treatment, the process shows a great improvement no matter whether in the degradation of dyes or photocurrent production under visible light. The TiO_2_ ALD layers effectively protect the nanostructures from corrosion and make electrons accessible to the electrolyte. The annealing treatment could adjust the noble metal NPs into a band gap-matched nanoscale. The rational design of nanocomposites helps make the photocatalytic performance of TiO_2_ in the visible wavelength region more efficient. Thus this method offers an efficient way to achieve the maximum usage of solar energy for photocatalytic degradation of organic pollutants in practical applications.

## Figures and Tables

**Table 1:** Degradation efficiency (%) under visible light (λ ≥ 420 nm) of MB.

ALD Cycle/Annealing Temperature	No Annealing Treatment	200 °C	300 °C	400 °C
No ALD	49.3	52.4	58.9	51.7
ALD1	63.3	63.3	74.7	61.4
ALD2	67.7	64.2	75.3	66.9
ALD3	62.6	67.6	65.3	67.3
ALD4	63.7	67.2	57	66.5

## Supplementary Materials

The supplementary materials are available online.
