# Supplementary material for: The Effect of Annealing Treatment and Atom Layer Deposition to Au/Pt Nanoparticles-Decorated TiO2 Nanorods as Photocatalysts
Source: Molecules. 2018 Feb 9;23(3):525. doi: 10.3390/molecules23030525 (PMC6017365; doi:10.3390/molecules23030525)
Supplement: Supplementary file 1 [file molecules-23-00525-s001.docx]

**Supplementary Materials**

**The Effect of Annealing Treatment and Atom Layer Deposition to Au/Pt Nanoparticles Decorated TiO_2_ Nanopillars as Photocatalysts**


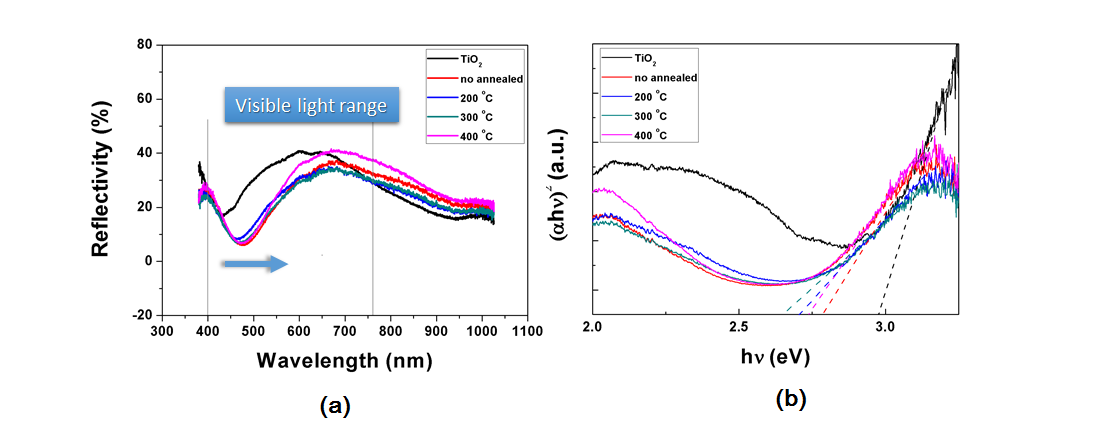


**Figure S1.** (a)UV-vis spectrum and (b) (αE_photon_)^2^ versus E_photon_ plots of all typical sample.
